# Atrial Natriuretic Peptide: A Molecular Target of Novel Therapeutic Approaches to Cardio-Metabolic Disease

**DOI:** 10.3390/ijms20133265

**Published:** 2019-07-02

**Authors:** Valentina Cannone, Aderville Cabassi, Riccardo Volpi, John C. Burnett

**Affiliations:** 1 Cardiorenal Research Laboratory, Circulatory Failure Division, Department of Cardiovascular Medicine, Mayo Clinic, Rochester, MN 55905, USA; 2 Division of Clinical Medicine, Department of Medicine and Surgery, University of Parma, 43126 Parma, Italy

**Keywords:** atrial natriuretic peptide, hypertension, heart failure, cardiometabolic disease, obesity, metabolic syndrome, cGMP, guanylyl cyclase receptor A, natriuretic peptides

## Abstract

Atrial natriuretic peptide (ANP) is a cardiac hormone with pleiotropic cardiovascular and metabolic properties including vasodilation, natriuresis and suppression of the renin-angiotensin-aldosterone system. Moreover, ANP induces lipolysis, lipid oxidation, adipocyte browning and ameliorates insulin sensitivity. Studies on ANP genetic variants revealed that subjects with higher ANP plasma levels have lower cardio-metabolic risk. In vivo and in humans, augmenting the ANP pathway has been shown to exert cardiovascular therapeutic actions while ameliorating the metabolic profile. MANP is a novel designer ANP-based peptide with greater and more sustained biological actions than ANP in animal models. Recent studies also demonstrated that MANP lowers blood pressure and inhibits aldosterone in hypertensive subjects whereas cardiometabolic properties of MANP are currently tested in an on-going clinical study in hypertension and metabolic syndrome. Evidence from in vitro, in vivo and in human studies support the concept that ANP and related pathway represent an optimal target for a comprehensive approach to cardiometabolic disease.

## 1. Introduction

The pioneering work of De Bold established the heart as an endocrine organ with the discovery of atrial natriuretic peptide (ANP), which is a 28 amino-acid cardiac hormone synthesized and secreted by cardiomyocytes in response to myocardial stretch [1]. Atrial natriuretic peptide is an endogenous ligand that activates the particulate guanylyl cyclase A receptor (GC–A) determining the production of the second messenger cyclic guanosine monophosphate (cGMP). Through protein kinase G and ion channels cGMP mediates ANP biological actions. Atrial natriuretic peptide also binds to the natriuretic peptide clearance receptor (NPR–C) that degrades ANP whereas the enzymes neprilysin and insulin degrading enzyme inactivate ANP via rapid peptide degradation. Since its discovery it was clear that ANP is a key regulator of cardiovascular volume and pressure homeostasis. Indeed, ANP induces vasodilation, natriuresis, diuresis and it counteracts the renin-angiotensin-aldosterone system (RAAS). In the kidney, ANP increases glomerular filtration rate by increasing afferent arteriolar dilation in addition to efferent arteriolar constriction [2]. At different levels of the nephron ANP inhibits water and sodium reabsorption. Atrial natriuretic peptide antagonizes the RAAS by inhibiting renin secretion and aldosterone production. Moreover, ANP has antihypertrophic and antifibrotic properties and genetic deletion of GC–A results in cardiac hypertrophy, and fibrosis [3,4,5,6,7,8]. Atrial natriuretic peptide also inhibits the sympathetic nervous system while increasing vagal activity [2]. In pathologic conditions such as heart failure excessive RAAS activation, volume overload and consequently augmented myocardial stretch, ANP production is increased. Through its unloading properties, ANP functions as a compensatory response to the altered cardiovascular homeostasis. As discussed later, ANP serves as a key target for novel therapies such as sacubitril/valsartan for the treatment of heart failure.

Hypertension, one of the main risk factors for the development of heart failure, is also considered to be the result of inappropriately high activity of RAAS and sympathetic nervous system along with sodium retention [9]. Importantly, in vivo and recent epidemiological studies reveal a key role exerted by ANP deficiency in the pathophysiology of this disease. In 1995, Smithies and coworkers reported the seminal observation that mice heterozygotes for the disruption of the ANP gene developed hypertension when fed a high sodium diet [10]. Several years later Macheret et al. reported that subjects with pre-hypertension have significantly lower ANP values compared to normotensive individuals [11]. Further, patients with hypertension do not display any increase in ANP levels that might exert a compensatory response to their cardiovascular pathological status. Probably the lack of adequate circulating ANP contributes to the onset of hypertension and increases the risk for cardiovascular diseases. In addition, recent epidemiological studies reveal an inverse relationship between aldosterone and ANP circulating levels in the general community and hypertensive subjects, with aldosterone being higher in the presence of lower ANP levels [12,13]. Interestingly, heart failure also appears to be a state of ANP deficiency based on a study conducted by Reginauld and coworkers [14]. In a cohort of 112 subjects with acute decompensated heart failure, 26% of patients did not show any compensatory increase in ANP levels. In this subgroup, circulating values of the second messenger, cGMP were also not increased.

An emerging concept is that the heart not only regulates blood pressure homeostasis but is also a regulator of whole body metabolism. Indeed, several studies revealed that ANP is a modulator of metabolism. More specifically, ANP induces lipid mobilization and oxidation and enhances insulin sensitivity [15]. Indeed, infusion of ANP in humans determines a lipolytic effect with an increase in plasma levels of glycerol and non-esterified fatty-acids regardless of their body mass index [16,17], while it also enhances energy expenditure [18]. In addition, intravenous administration of ANP results in an increase in plasma levels of adiponectin [19,20]. This cytokine, which is secreted by adipocytes and cardiomyocytes, possesses cardioprotective properties and regulatory effects on glucose and lipid metabolism ameliorating insulin-sensitivity [21,22,23,24]. In vitro studies in subcutaneous adipocytes also showed that ANP inhibits the production of inflammatory cytokines involved in obesity-related inflammatory state and insulin resistance [25]. In rodents, ANP induces browning of adipocytes along with mitochondrial biogenesis [26], whereas in human skeletal muscle, the cardiac hormone increases mitochondrial oxidative metabolism and fat oxidation [27]. These important studies on ANP metabolic action are underscored by epidemiological studies reporting a relationship between ANP and metabolic diseases. Circulating levels of ANP are lower in obese compared to lean individuals [28] and are inversely related to each metabolic criterion of metabolic syndrome [29]. Low levels of ANP are also predictive of future development of diabetes [30]. The mechanisms underlying these associations might be related to an insulin/glucose-mediated regulation of the GC–A and NPR–C. Recent in vitro studies showed that insulin upregulates NPR–C expression in adipocytes in a glucose-dependent manner with an increased expression observed in the presence of higher glucose concentrations [31]. In humans, higher fasting levels of insulin and insulin resistance measured by homeostatic model assessment for insulin resistance (HOMA) positively correlate with adipose tissue NPRC expression. Indeed, expression of NPR–C is augmented, whereas GC–A is significantly decreased in adipose tissue of obese compared to normal weight subjects [32,33]. The ratio GC–A/NPR–C increases by losing weight and ameliorating insulin-sensitivity [34]. A similar pattern is observed for skeletal muscle with GC–A expression, which is inversely related to fat content, body mass index, fasting plasma insulin levels and insulin resistance, whereas NPR–C is upregulated in obese individuals with impaired glucose tolerance and type 2 diabetes [35]. Coue and coworkers provided new insights into the metabolic actions of ANP and reported that a potential mechanism through which ANP favors insulin sensitivity involves increasing glucose uptake in adipocytes [36]. Most interesting was the observation that the effect is attenuated in obesity, providing a further possible cross-talk between obesity and insulin resistance.

When these studies in vivo, in vitro and in humans are taken together, they show the important role played by ANP in cardiovascular and metabolic homeostasis, highlighting how this cardiac hormone could be an important therapeutic target in cardio-metabolic disease.

## 2. Atrial Natriuretic Peptide Genetic Variants

The natriuretic peptide precursor A gene (*NPPA*) is located on chromosome 1 and encodes the pre-pro-hormone from which ANP is obtained after cleavage. Over the last two decades key studies in humans have investigated the phenotype associated with single nucleotide polymorphisms of *NPPA* and revealed clinical findings, which further support the important role of ANP in defining the cardiovascular and metabolic phenotype.

The single nucleotide polymorphism rs5068, located in the 3′-UTR of *NPPA*, is associated with higher circulating levels of ANP [37,38,39,40]. In 2009, Newton-Cheh and coworkers showed that the minor G allele of rs5068 is related to higher plasma levels of ANP in general community-cohorts of whites from the United States and Northern Europe [37]. In line with ANP biological properties, the minor G allele is also associated with lower blood pressure and risk of hypertension. Cannone et al. investigated not only the cardiovascular but also the metabolic phenotype associated with this genetic variant in a general population of whites from the United States [38]. The carriers of rs5068 G minor allele, who have higher circulating levels of ANP and lower systolic blood pressure values, also have lower body mass index and waist circumference. A key finding of this genetic study was the lower prevalence of obesity and metabolic syndrome among the carriers of the minor allele. In addition, protective plasma high-density lipoprotein cholesterol was higher whereas C-reactive protein levels were lower in the AG/GG genotypes. Importantly, the association between rs5068 minor G allele and a clinical phenotype characterized by lower cardio-metabolic risk was replicated in a general community from the Mediterranean island of Sicily [41]. In non-diabetic Northern Europeans, the rs5068 minor G allele is associated with lower prevalence of left ventricular hypertrophy and decreased risk of developing diabetes in a 14-year follow up analysis [42,43]. The phenotype associated with rs5068 genotypes was also analyzed in African Americans and of note, subjects who are carriers of the G allele have lower triglycerides and insulin levels as well as higher high-density lipoprotein cholesterol [44]. Diabetes and metabolic syndrome are less prevalent among the AG/GG genotypes. The mechanism underlying the associations between rs5068 minor G allele and higher circulating levels of ANP was investigated by Arora et al. in an interesting study showing that this single nucleotide polymorphism does not allow micro-RNA 425 to attach to the complementary sequence and exert its inhibitory effect, resulting in a higher production of ANP [45].

While higher levels of ANP are protective, the emerging concept is that subjects who are exposed to lower circulating levels of ANP also have higher cardio-metabolic risk. Indeed, in a study aimed to identify genetic determinants of ANP plasma levels, Pereira et al. revealed that the ANP genetic variant rs5063 is associated with lower ANP levels, and the carriers of this single nucleotide polymorphism have higher diastolic blood pressure and risk of stroke [46].

Single nucleotide polymorphisms as rs5068 and rs5063, which are associated with variations of ANP circulating levels, provide the opportunity to investigate the phenotype related to a life-long exposure to higher or lower ANP plasma levels. The clinical characteristics observed in the carriers of rs5068 and rs5063 are consistent with the blood pressure lowering, lipolytic and insulin sensitizing effect of ANP and further support the concept of ANP as a therapeutic strategy in the treatment of cardio-metabolic disease.

## 3. Atrial Natriuretic Peptide as a Therapeutic for Cardio-Metabolic Disease

Metabolic Syndrome consists of several cardiovascular risk factors including elevated blood pressure, abdominal obesity, dyslipidemia and impaired fasting glucose [47]. Each factor is independently associated with the development of atherosclerotic cardiovascular disease and type 2 diabetes. Metabolic syndrome does not appear to be determined by a single cause but precipitated by two main underlying pathological conditions, which are abdominal obesity and insulin resistance. In the United States general adult population, the prevalence of the metabolic syndrome (including those with diabetes mellitus) is approximately 34% whereas diabetes mellitus and obesity, which also represent major risk factors for the development of cardiovascular disease, have a prevalence of 13% and 40%, respectively [48]. Hypertension is widely prevalent in the United States affecting around 32% of the adult population and represents one of the main features of metabolic syndrome [49,50]. If the most recent 2017 American College of Cardiology/American Heart Association guidelines for hypertension (defined as taking antihypertensive medication, or a systolic pressure ≥130 mmHg and/or a diastolic pressure ≥80 mmHg) are applied the prevalence raises to 46% based on the National Health and Nutrition Examination Survey data from 2011 to 2014 [51,52]. Hypertension and metabolic disease are closely interrelated, indeed, hypertension is present in 77% of the patients affected by metabolic syndrome and abdominal obesity is considered one of the most important risk factors for the development of hypertension [53,54]. One of the main pathophysiological pathways that link obesity and hypertension is the excessive activation of the sympathetic nervous system and the RAAS, which leads to an increase in sodium and water retention [55]. Both hypertension and metabolic syndrome represent a significant risk factor for the development of cardiovascular disease when considered individually [56,57,58]. If they coexist, the risk for cardiovascular events is further amplified, being almost double [59]. Heart failure affects 6.2 million (prevalence, 2.2%) of United States adults and hypertension, metabolic syndrome, obesity along with diabetes represent all well-known risk factors for its onset [48].

Previous epidemiological and physiological studies illustrated above support the concept that hypertension and heart failure as well as obesity and metabolic syndrome are conditions characterized by ANP deficiency. Moreover, with its favorable cardiovascular and metabolic properties ANP represents an appealing target for a combined approach to cardio-metabolic disease. In Japan, ANP in its recombinant form of carperitide has been successfully used for many years for the treatment of heart failure [60]. Infusion of carperitide has been shown to improve clinical conditions and degree of dyspnea in subjects with acute heart failure [61,62]. An 18-month follow-up analysis also revealed that low-dose carperitide infused for 72 h as the initial treatment in addition to standard therapy is associated with lower risk of re-hospitalization and mortality [63]. In 2014, the PARADIGM Trial demonstrated the beneficial therapeutic effect of sacubitril/valsartan (LCZ696), a combined angiotensin II receptor-neprilysin inhibitor, in subjects with heart failure with reduced ejection fraction [64]. Chronic heart failure patients who received sacubitril/valsartan as opposed to enalapril in addition to recommended standard therapy had lower risk of mortality and re-hospitalization. The benefit of LCZ696 included also an improvement in heart failure symptoms and physical limitations. When tested in the setting of acutely decompensated heart failure, sacubitril/valsartan resulted in a significant decrease in N-terminal pro-B-type natriuretic peptide as a marker of neurohormonal activation and hemodynamic stress [65]. A key mechanism of action of sacubitril/valsartan is the inhibition of neprilysin. Being neprilysin involved in the degradation of several peptides and related pathways including ANP, two recent interesting studies investigated the effect of sacubitril on the enzyme substrates [66,67]. Importantly, both studies report a significant and sustained increase of ANP in subjects receiving sacubitril/valsartan, which further support the concept that augmenting ANP circulating levels has a beneficial effect in the long-term treatment of cardiovascular diseases. Sacubitril/valsartan appears also to be more effective than valsartan alone in the treatment of hypertension [68]. In a multicenter, randomized, double-blind, cross-over study in Asian subjects with salt-sensitive hypertension, the use of valsartan/sacubitril lead to a greater reduction in both office and 24 h-ambulatory blood pressure over a 28 day period. The blood pressure lowering effect was accompanied by an acute significant increase of natriuresis and diuresis on the first day of therapy with angiotensin II receptor-neprilysin inhibitor. The metabolic effect of sacubitril/valsartan was tested in patients with cardio-metabolic disease. In a post-hoc analysis of the PARADIGM trial, subjects with heart failure and diabetes or HbA1c ≥ 6.5% who were randomized to sacubitril/valsartan had a better glycemic control than subjects receiving enalapril [69]. Over the three-year follow-up HbA1c concentrations and new use of insulin or oral antihyperglycaemic agents were all significantly lower when compared to the control group. In a cohort of subjects with hypertension and obesity, eight-week treatment with sacubitril/valasartan increased insulin-sensitivity and abdominal subcutaneous adipose tissue lipolysis [70].

These studies discussed above clearly support the therapeutic potential of ANP in cardio-metabolic disease. However, ANP has a half-life of 2–5 min, which renders this cardiac peptide unsuitable for a single or twice daily administration. Conversely, MANP is a 40 amino acid peptide with a 12 amino acid extension to the carboxyl-terminus of ANP and has a half-life of 45 min. MANP was engineered at Mayo Clinic to represent a novel GC-A activator with biological actions that are superior to native ANP. Indeed, the novel 12 amino acid extension on the carboxyl-terminus results in MANP being highly resistant to enzymatic degradation by both neprilysin and insulin-degrading enzyme, but does not alter the high affinity for GC–A [71,72]. When compared to native ANP intravenous infusion of MANP in normal canines resulted in a greater activation of the second messenger cGMP and consequent greater and more sustained blood pressure lowering effect, increase in renal blood flow and glomerular filtration rate [73]. Diuresis and natriuresis were augmented in addition to a greater and more sustained inhibition of angiotensin II and aldosterone. MANP was also tested in a canine model of hypertension obtained by continuous infusion of angiotensin II [74]. Intravenous administration of MANP determined a significant decrease in mean arterial pressure, which was sustained up to two hours after MANP infusion. Systemic vascular resistance decreased along with pulmonary capillary wedge pressure, pulmonary artery pressure and right atrial pressure. In spite of the systemic blood pressure lowering effect, renal blood flow and glomerular filtration rate increased in addition to a marked rise in water and sodium excretion. Proximal and distal sodium reabsorption of sodium decreased during MANP infusion and for the following hour. The biological properties of MANP, which emerged from these two in vivo studies, made MANP an unprecedented therapeutic candidate for being tested in a large animal model of heart failure with hypertension [75]. Indeed, MANP was infused for 45 min and its effects were compared with nitroglycerin, which is a therapeutic agent commonly used in the clinical setting of heart failure. MANP administration resulted in a greater and sustained increase in the plasma levels of cGMP whereas nitroglycerin did not change circulating levels of the second messenger. While both MANP and nitroglycerin lowered mean arterial pressure, MANP effect was more sustained in time. Both compounds reduced systemic and renal vascular resistance in addition to pulmonary capillary wedge pressure, pulmonary and right atrial pressures. Importantly, only MANP was found to have a renal hemodynamic effect inducing a significant increase in renal blood flow, which continued for 120 min after the administration. Glomerular filtration rate, diuresis and natriuresis were also markedly increased during the infusion of MANP and for the following 60 min. In contrast to nitroglycerin, which did not modify aldosterone levels, during MANP administration circulating aldosterone values were significantly lower than baseline.

Recent studies further tested the cardiovascular and metabolic actions of MANP in vitro and in vivo. When human subcutaneous and visceral pre-adipocytes were incubated with MANP the cGMP pathway was activated inducing a significant production of the second messenger [76]. In normal Sprague–Dawley rats, MANP acute infusion significantly decreased mean arterial pressure while increasing non-esterified fatty acids, which is a marker of lipolysis. On-going studies are also testing MANP properties in a rodent model of hypertension and metabolic syndrome. Acute intravenous administration of MANP reduced blood pressure and induced an increase in circulating levels of adiponectin. Importantly, these in vitro and in vivo findings highlight how MANP not only retains cardiovascular and metabolic properties of native ANP but also might display greater and more sustained biological actions.

A first in human study of MANP was recently completed [77]. MANP was administered to humans with stable hypertension withdrawn from anti-hypertensive medications for two weeks. The study was designed as a single ascending dose administered subcutaneously as a single injection. The goal was to determine the maximal tolerated dose as well as to establish safety and tolerability. Three different doses (1, 2.5 and 5 µg/kg) of MANP were tested in three different groups. Each group included four subjects receiving MANP and one subject receiving a placebo in addition to their usual antihypertensive therapy. Blood pressure was assessed over a 24 h period. Preliminary data demonstrated that MANP was well tolerated with no serious adverse effects. Mild adverse events included mild headache; transient light-headedness and transient orthostatic vasovagal syncope lasting for 7 s in one subject. No significant changes in laboratory values or electrocradiogram from baseline were observed. Blood pressure was reduced with all doses. Specifically, the maximal systolic blood pressure reduction was 20 mmHg (with 5.0 μg/kg); maximal duration of systolic blood pressure reduction was 24 hr (with 2.5 and 5.0 μg/kg); maximal diastolic blood pressure reduction was 12 mmHg (with 2.5 and 5.0 μg/kg) and maximal duration of diastolic blood pressure reduction was 24 hr (with 5.0 μg/kg). Cyclic GMP plasma levels were increased whereas aldosterone circulating levels were descreased with all MANP doses administered. Importantly, an on-going clinical study is currently evaluating the cardiovascular and metabolic actions of MANP in subjects with hypertension and metabolic syndrome with the primary goal of assessing the potential therapeutic effect of MANP in cardio-metabolic disease. To date, no studies have compared the recombinant form of human ANP, Carperitide, with MANP. Future assessments might evaluate the cardio-metabolic effects of the two compounds in the clinical setting of heart failure.

**Figure 1:**
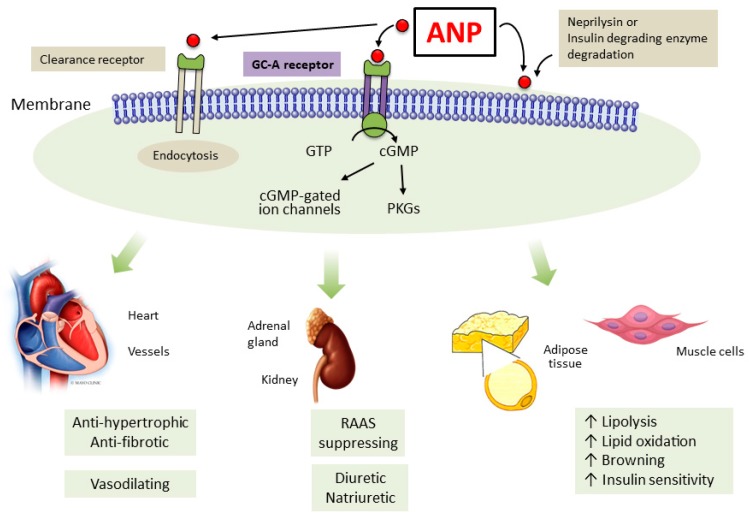
Atrial natriuretic peptide binds to the guanylyl cyclase A receptor (GC–A) resulting in the production of the second messenger cyclic guanosine monophosphate (cGMP). Protein kinase G (PKG) and cGMP-gated ion channels are then activated and mediate ANP biological actions, which are summarized in the figure.

In conclusion, ANP is a cardiac hormone with pleiotropic biological actions (Figure 1). In vivo, in vitro, epidemiological and in human studies support the concept that augmenting ANP and related pathways might be a successful strategy in the treatment of cardio-metabolic disease. MANP, a novel designer ANP-based peptide, which has been tested and is currently being tested in clinical trials, represents a potentially safe, effective and comprehensive therapeutic approach to cardio-metabolic dysfunction.

## Abbreviations

ANP	Atrial natriuretic peptide
cGMP	Cyclic guanosine monophosphate
GC–A	Guanylyl cyclase A receptor
HOMA	Homeostatic model assessment for insulin resistance
NPPA	Natriuretic peptide precursor A gene
NPR–C	Natriuretic peptide clearance receptor
RAAS	Renin-angiotensin-aldosterone system

## References

[1] Garbers D.L., Chrisman T.D., Wiegn P., Katafuchi T., Albanesi J.P., Bielinski V., Barylko B., Redfield M.M., Burnett J.C. (2006). Membrane guanylyl cyclase receptors: An update. Trends Endocrinol. Metab..

[2] Volpe M., Carnovali M., Mastromarino V. (2016). The natriuretic peptides system in the pathophysiology of heart failure: From molecular basis to treatment. Clin. Sci. (Lond.).

[3] Patel J.B., Valencik M.L., Pritchett A.M., Burnett J.C., McDonald J.A., Redfield M.M. (2005). Cardiac-specific attenuation of natriuretic peptide A receptor activity accentuates adverse cardiac remodeling and mortality in response to pressure overload. Am. J. Physiol. Heart Circ. Physiol..

[4] Holtwick R., van Eickels M., Skryabin B.V., Baba H.A., Bubikat A., Begrow F., Schneider M.D., Garbers D.L., Kuhn M. (2003). Pressure-independent cardiac hypertrophy in mice with cardiomyocyte-restricted inactivation of the atrial natriuretic peptide receptor guanylyl cyclase-A. J. Clin. Investig..

[5] Calderone A., Thaik C.M., Takahashi N., Chang D.L., Colucci W.S. (1998). Nitric oxide, atrial natriuretic peptide, and cyclic GMP inhibit the growth-promoting effects of norepinephrine in cardiac myocytes and fibroblasts. J. Clin. Investig..

[6] Vellaichamy E., Kaur K., Pandey K.N. (2007). Enhanced activation of pro-inflammatory cytokines in mice lacking natriuretic peptide receptor-A. Peptides.

[7] Wang D., Oparil S., Feng J.A., Li P., Perry G., Chen L.B., Dai M., John S.W., Chen Y.F. (2003). Effects of pressure overload on extracellular matrix expression in the heart of the atrial natriuretic peptide-null mouse. Hypertension.

[8] Oliver P.M., Fox J.E., Kim R., Rockman H.A., Kim H.S., Reddick R.L., Pandey K.N., Milgram S.L., Smithies O., Maeda N. (1997). Hypertension, cardiac hypertrophy, and sudden death in mice lacking natriuretic peptide receptor A. Proc. Natl. Acad. Sci. USA.

[9] Goodfriend T.L., Calhoun D.A. (2004). Resistant hypertension, obesity, sleep apnea, and aldosterone: Theory and therapy. Hypertension.

[10] John S.W., Krege J.H., Oliver P.M., Hagaman J.R., Hodgin J.B., Pang S.C., Flynn T.G., Smithies O. (1995). Genetic decreases in atrial natriuretic peptide and salt-sensitive hypertension. Science.

[11] Macheret F., Heublein D., Costello-Boerrigter L.C., Boerrigter G., McKie P., Bellavia D., Mangiafico S., Ikeda Y., Bailey K., Scott C.G. (2012). Human hypertension is characterized by a lack of activation of the antihypertensive cardiac hormones ANP and BNP. J. Am. Coll. Cardiol..

[12] Buglioni A., Cannone V., Cataliotti A., Sangaralingham S.J., Heublein D.M., Scott C.G., Bailey K.R., Rodeheffer R.J., Dessi-Fulgheri P., Sarzani R. (2015). Circulating aldosterone and natriuretic peptides in the general community: Relationship to cardiorenal and metabolic disease. Hypertension.

[13] Cannone V., Buglioni A., Sangaralingham S.J., Scott C., Bailey K.R., Rodeheffer R., Redfield M.M., Sarzani R., Burnett J.C. (2018). Aldosterone, hypertension, and antihypertensive therapy: Ifrom a general population. Mayo. Clin. Proc..

[14] Reginauld S.H., Cannone V., Iyer S., Scott C., Baily K., Schaefer J., Chen Y., Sangaralingham S.J., Burnett J.C. (2019). Differential regulation of ANP and BNP in acute decompensated heart failure - deficiency of ANP. J. Am. Coll. Cardiol..

[15] Coue M., Moro C. (2016). Natriuretic peptide control of energy balance and glucose homeostasis. Biochimie.

[16] Birkenfeld A.L., Boschmann M., Moro C., Adams F., Heusser K., Franke G., Berlan M., Luft F.C., Lafontan M., Jordan J. (2005). Lipid mobilization with physiological atrial natriuretic peptide concentrations in humans. J. Clin. Endocrinol. Metab..

[17] Galitzky J., Sengenes C., Thalamas C., Marques M.A., Senard J.M., Lafontan M., Berlan M. (2001). The lipid-mobilizing effect of atrial natriuretic peptide is unrelated to sympathetic nervous system activation or obesity in young men. J. Lipid Res..

[18] Birkenfeld A.L., Budziarek P., Boschmann M., Moro C., Adams F., Franke G., Berlan M., Marques M.A., Sweep F.C., Luft F.C. (2008). Atrial natriuretic peptide induces postprandial lipid oxidation in humans. Diabetes.

[19] Tsukamoto O., Fujita M., Kato M., Yamazaki S., Asano Y., Ogai A., Okazaki H., Asai M., Nagamachi Y., Maeda N. (2009). Natriuretic peptides enhance the production of adiponectin in human adipocytes and in patients with chronic heart failure. J. Am. Coll. Cardiol..

[20] Birkenfeld A.L., Boschmann M., Engeli S., Moro C., Arafat A.M., Luft F.C., Jordan J. (2012). Atrial natriuretic peptide and adiponectin interactions in man. PLoS ONE.

[21] Maeda N., Shimomura I., Kishida K., Nishizawa H., Matsuda M., Nagaretani H., Furuyama N., Kondo H., Takahashi M., Arita Y. (2002). Diet-induced insulin resistance in mice lacking adiponectin/ACRP30. Nat. Med..

[22] Shibata R., Ouchi N., Ito M., Kihara S., Shiojima I., Pimentel D.R., Kumada M., Sato K., Schiekofer S., Ohashi K. (2004). Adiponectin-mediated modulation of hypertrophic signals in the heart. Nat. Med..

[23] Shibata R., Sato K., Pimentel D.R., Takemura Y., Kihara S., Ohashi K., Funahashi T., Ouchi N., Walsh K. (2005). Adiponectin protects against myocardial ischemia-reperfusion injury through AMPK- and COX-2-dependent mechanisms. Nat. Med..

[24] Costello-Boerrigter L.C., Burnett J.C. (2009). A new role for the natriuretic peptides: Metabolic regulators of the adipocyte. J. Am. Coll. Cardiol..

[25] Moro C., Klimcakova E., Lolmede K., Berlan M., Lafontan M., Stich V., Bouloumie A., Galitzky J., Arner P., Langin D. (2007). Atrial natriuretic peptide inhibits the production of adipokines and cytokines linked to inflammation and insulin resistance in human subcutaneous adipose tissue. Diabetologia.

[26] Bordicchia M., Liu D., Amri E.Z., Ailhaud G., Dessi-Fulgheri P., Zhang C., Takahashi N., Sarzani R., Collins S. (2012). Cardiac natriuretic peptides act via p38 MAPK to induce the brown fat thermogenic program in mouse and human adipocytes. J. Clin. Investig..

[27] Engeli S., Birkenfeld A.L., Badin P.M., Bourlier V., Louche K., Viguerie N., Thalamas C., Montastier E., Larrouy D., Harant I. (2012). Natriuretic peptides enhance the oxidative capacity of human skeletal muscle. J. Clin. Investig..

[28] Wang T.J., Larson M.G., Levy D., Benjamin E.J., Leip E.P., Wilson P.W., Vasan R.S. (2004). Impact of obesity on plasma natriuretic peptide levels. Circulation.

[29] Wang T.J., Larson M.G., Keyes M.J., Levy D., Benjamin E.J., Vasan R.S. (2007). Association of plasma natriuretic peptide levels with metabolic risk factors in ambulatory individuals. Circulation.

[30] Magnusson M., Jujic A., Hedblad B., Engstrom G., Persson M., Struck J., Morgenthaler N.G., Nilsson P., Newton-Cheh C., Wang T.J. (2012). Low plasma level of atrial natriuretic peptide predicts development of diabetes: The prospective Malmo Diet and Cancer study. J. Clin. Endocrinol. Metab..

[31] Bordicchia M., Ceresiani M., Pavani M., Minardi D., Polito M., Wabitsch M., Cannone V., Burnett J.C., Dessi-Fulgheri P., Sarzani R. (2016). Insulin/glucose induces natriuretic peptide clearance receptor in human adipocytes: A metabolic link with the cardiac natriuretic pathway. Am. J. Physiol. Regul. Integr. Comp. Physiol..

[32] Ryden M., Backdahl J., Petrus P., Thorell A., Gao H., Coue M., Langin D., Moro C., Arner P. (2016). Impaired atrial natriuretic peptide-mediated lipolysis in obesity. Int. J. Obes. (Lond.).

[33] Verboven K., Hansen D., Moro C., Eijnde B.O., Hoebers N., Knol J., Bouckaert W., Dams A., Blaak E.E., Jocken J.W. (2016). Attenuated atrial natriuretic peptide-mediated lipolysis in subcutaneous adipocytes of obese type 2 diabetic men. Clin. Sci. (Lond.).

[34] Kovacova Z., Tharp W.G., Liu D., Wei W., Xie H., Collins S., Pratley R.E. (2016). Adipose tissue natriuretic peptide receptor expression is related to insulin sensitivity in obesity and diabetes. Obesity (Silver Spring).

[35] Coue M., Badin P.M., Vila I.K., Laurens C., Louche K., Marques M.A., Bourlier V., Mouisel E., Tavernier G., Rustan A.C. (2015). Defective natriuretic peptide receptor signaling in skeletal muscle links obesity to type 2 diabetes. Diabetes.

[36] Coue M., Barquissau V., Morigny P., Louche K., Lefort C., Mairal A., Carpene C., Viguerie N., Arner P., Langin D. (2018). Natriuretic peptides promote glucose uptake in a cGMP-dependent manner in human adipocytes. Sci. Rep..

[37] Newton-Cheh C., Larson M.G., Vasan R.S., Levy D., Bloch K.D., Surti A., Guiducci C., Kathiresan S., Benjamin E.J., Struck J. (2009). Association of common variants in NPPA and NPPB with circulating natriuretic peptides and blood pressure. Nat. Genet..

[38] Cannone V., Boerrigter G., Cataliotti A., Costello-Boerrigter L.C., Olson T.M., McKie P.M., Heublein D.M., Lahr B.D., Bailey K.R., Averna M. (2011). A genetic variant of the atrial natriuretic peptide gene is associated with cardiometabolic protection in the general community. J. Am. Coll. Cardiol..

[39] Cannone V., Barlera S., Pileggi S., Masson S., Franzosi M.G., Latini R., Scardulla C., Clemenza F., Maggioni A.P., Nicolosi G.L. (2014). The Anp genetic variant Rs5068 and circulating levels of natriuretic peptides in patients with chronic heart failure. Int. J. Cardiol..

[40] Ellis K.L., Newton-Cheh C., Wang T.J., Frampton C.M., Doughty R.N., Whalley G.A., Ellis C.J., Skelton L., Davis N., Yandle T.G. (2011). Association of genetic variation in the natriuretic peptide system with cardiovascular outcomes. J. Mol. Cell. Cardiol..

[41] Cannone V., Cefalu A.B., Noto D., Scott C.G., Bailey K.R., Cavera G., Pagano M., Sapienza M., Averna M.R., Burnett J.C. (2013). The atrial natriuretic peptide genetic variant rs5068 is associated with a favorable cardiometabolic phenotype in a Mediterranean population. Diabetes Care..

[42] Jujic A., Leosdottir M., Ostling G., Gudmundsson P., Nilsson P.M., Melander O., Magnusson M. (2013). A genetic variant of the atrial natriuretic peptide gene is associated with left ventricular hypertrophy in a non-diabetic population--the Malmo preventive project study. BMC Med. Genet..

[43] Jujic A., Nilsson P.M., Engstrom G., Hedblad B., Melander O., Magnusson M. (2014). Atrial natriuretic peptide and type 2 diabetes development--biomarker and genotype association study. PLoS ONE.

[44] Cannone V., Scott C.G., Decker P.A., Larson N.B., Palmas W., Taylor K.D., Wang T.J., Gupta D.K., Bielinski S.J., Burnett J.C. (2017). A favorable cardiometabolic profile is associated with the G allele of the genetic variant rs5068 in African Americans: The Multi-Ethnic Study of Atherosclerosis (MESA). PLoS ONE.

[45] Arora P., Wu C., Khan A.M., Bloch D.B., Davis-Dusenbery B.N., Ghorbani A., Spagnolli E., Martinez A., Ryan A., Tainsh L.T. (2013). Atrial natriuretic peptide is negatively regulated by microRNA-425. J. Clin. Investig..

[46] Pereira N.L., Tosakulwong N., Scott C.G., Jenkins G.D., Prodduturi N., Chai Y., Olson T.M., Rodeheffer R.J., Redfield M.M., Weinshilboum R.M. (2015). Circulating atrial natriuretic peptide genetic association study identifies a novel gene cluster associated with stroke in whites. Circ. Cardiovasc. Genet..

[47] Eckel R.H., Alberti K.G., Grundy S.M., Zimmet P.Z. (2010). The metabolic syndrome. Lancet.

[48] Benjamin E.J., Muntner P., Alonso A., Bittencourt M.S., Callaway C.W., Carson A.P., Chamberlain A.M., Chang A.R., Cheng S., Das S.R. (2019). Heart disease and stroke statistics-2019 update: A report from the American Heart Association. Circulation.

[49] Yoon S.S., Carroll M.D., Fryar C.D. (2015). Hypertension prevalence and control among adults: United States, 2011-2014. NCHS Data Brief..

[50] Egan B.M., Li J., Hutchison F.N., Ferdinand K.C. (2014). Hypertension in the United States, 1999 to 2012: Progress toward Healthy People 2020 goals. Circulation.

[51] Whelton P.K., Carey R.M., Aronow W.S., Casey D.E., Collins K.J., Dennison Himmelfarb C., DePalma S.M., Gidding S., Jamerson K.A., Jones D.W. (2018). 2017 ACC/AHA/AAPA/ABC/ACPM/AGS/APhA/ASH/ASPC/NMA/PCNA Guideline for the prevention, detection, evaluation, and management of high blood pressure in adults: A report of the American College of Cardiology/American Heart Association task force on clinical practice guidelines. Hypertension.

[52] Muntner P., Carey R.M., Gidding S., Jones D.W., Taler S.J., Wright J.T., Whelton P.K. (2018). Potential US population impact of the 2017 ACC/AHA high blood pressure guideline. Circulation.

[53] Mozaffarian D., Benjamin E.J., Go A.S., Arnett D.K., Blaha M.J., Cushman M., de Ferranti S., Despres J.P., Fullerton H.J., Howard V.J. (2015). Heart disease and stroke statistics--2015 update: A report from the American Heart Association. Circulation.

[54] Sironi A.M., Gastaldelli A., Mari A., Ciociaro D., Positano V., Buzzigoli E., Ghione S., Turchi S., Lombardi M., Ferrannini E. (2004). Visceral fat in hypertension: Influence on insulin resistance and beta-cell function. Hypertension.

[55] Rahmouni K., Correia M.L., Haynes W.G., Mark A.L. (2005). Obesity-associated hypertension: New insights into mechanisms. Hypertension.

[56] Chobanian A.V., Bakris G.L., Black H.R., Cushman W.C., Green L.A., Izzo J.L., Jones D.W., Materson B.J., Oparil S., Wright J.T. (2003). Seventh report of the Joint National Committee on Prevention, Detection, Evaluation, and Treatment of High Blood Pressure. Hypertension.

[57] Ford E.S. (2005). Risks for all-cause mortality, cardiovascular disease, and diabetes associated with the metabolic syndrome: A summary of the evidence. Diabetes Care.

[58] Galassi A., Reynolds K., He J. (2006). Metabolic syndrome and risk of cardiovascular disease: A meta-analysis. Am. J. Med..

[59] Schillaci G., Pirro M., Vaudo G., Gemelli F., Marchesi S., Porcellati C., Mannarino E. (2004). Prognostic value of the metabolic syndrome in essential hypertension. J. Am. Coll. Cardiol..

[60] Saito Y. (2010). Roles of atrial natriuretic peptide and its therapeutic use. J. Cardiol..

[61] Suwa M., Seino Y., Nomachi Y., Matsuki S., Funahashi K. (2005). Multicenter prospective investigation on efficacy and safety of carperitide for acute heart failure in the ‘real world’ of therapy. Circ. J..

[62] Nomura F., Kurobe N., Mori Y., Hikita A., Kawai M., Suwa M., Okutani Y. (2008). Multicenter prospective investigation on efficacy and safety of carperitide as a first-line drug for acute heart failure syndrome with preserved blood pressure: COMPASS: Carperitide Effects Observed Through Monitoring Dyspnea in Acute Decompensated Heart Failure Study. Circ. J..

[63] Hata N., Seino Y., Tsutamoto T., Hiramitsu S., Kaneko N., Yoshikawa T., Yokoyama H., Tanaka K., Mizuno K., Nejima J. (2008). Effects of carperitide on the long-term prognosis of patients with acute decompensated chronic heart failure: The PROTECT multicenter randomized controlled study. Circ. J..

[64] McMurray J.J., Packer M., Desai A.S., Gong J., Lefkowitz M.P., Rizkala A.R., Rouleau J.L., Shi V.C., Solomon S.D., Swedberg K. (2014). Angiotensin-neprilysin inhibition versus enalapril in heart failure. N. Engl. J. Med..

[65] Velazquez E.J., Morrow D.A., DeVore A.D., Duffy C.I., Ambrosy A.P., McCague K., Rocha R., Braunwald E., Investigators P.-H. (2019). Angiotensin-neprilysin inhibition in acute decompensated heart failure. N. Engl. J. Med..

[66] Nougue H., Pezel T., Picard F., Sadoune M., Arrigo M., Beauvais F., Launay J.M., Cohen-Solal A., Vodovar N., Logeart D. (2019). Effects of sacubitril/valsartan on neprilysin targets and the metabolism of natriuretic peptides in chronic heart failure: A mechanistic clinical study. Eur. J. Heart Fail..

[67] Ibrahim N.E., McCarthy C.P., Shrestha S., Gaggin H.K., Mukai R., Szymonifka J., Apple F.S., Burnett J.C., Iyer S., Januzzi J.L. (2019). Effect of neprilysin inhibition on various natriuretic peptide assays. J. Am. Coll. Cardiol..

[68] Wang T.D., Tan R.S., Lee H.Y., Ihm S.H., Rhee M.Y., Tomlinson B., Pal P., Yang F., Hirschhorn E., Prescott M.F. (2017). Effects of sacubitril/valsartan (LCZ696) on natriuresis, diuresis, blood pressures, and NT-proBNP in salt-sensitive hypertension. Hypertension.

[69] Seferovic J.P., Claggett B., Seidelmann S.B., Seely E.W., Packer M., Zile M.R., Rouleau J.L., Swedberg K., Lefkowitz M., Shi V.C. (2017). Effect of sacubitril/valsartan versus enalapril on glycaemic control in patients with heart failure and diabetes: A post-hoc analysis from the PARADIGM-HF trial. Lancet. Diabetes Endocrinol..

[70] Jordan J., Stinkens R., Jax T., Engeli S., Blaak E.E., May M., Havekes B., Schindler C., Albrecht D., Pal P. (2017). Improved insulin sensitivity with angiotensin receptor neprilysin inhibition in individuals with obesity and hypertension. Clin. Pharmacol. Ther..

[71] Dickey D.M., Yoder A.R., Potter L.R. (2009). A familial mutation renders atrial natriuretic Peptide resistant to proteolytic degradation. J. Biol. Chem..

[72] Ralat L.A., Guo Q., Ren M., Funke T., Dickey D.M., Potter L.R., Tang W.J. (2011). Insulin-degrading enzyme modulates the natriuretic peptide-mediated signaling response. J. Biol. Chem..

[73] McKie P.M., Cataliotti A., Huntley B.K., Martin F.L., Olson T.M., Burnett J.C. (2009). A human atrial natriuretic peptide gene mutation reveals a novel peptide with enhanced blood pressure-lowering, renal-enhancing, and aldosterone-suppressing actions. J. Am. Coll. Cardiol..

[74] McKie P.M., Cataliotti A., Boerrigter G., Chen H.H., Sangaralingham S.J., Martin F.L., Ichiki T., Burnett J.C. (2010). A novel atrial natriuretic peptide based therapeutic in experimental angiotensin II mediated acute hypertension. Hypertension.

[75] McKie P.M., Cataliotti A., Ichiki T., Sangaralingham S.J., Chen H.H., Burnett J.C. (2014). M-atrial natriuretic peptide and nitroglycerin in a canine model of experimental acute hypertensive heart failure: Differential actions of 2 cGMP activating therapeutics. J. Am. Heart Assoc..

[76] Cannone V., Huntley B.K., Heublein D.M., Sandberg S.M., Harders G.E., Sangaralingham J.S., Martin F.L., Burnett J.C. (2012). MANP: A novel designer natriuretic peptide for cardiometabolic disease. J. Card Fail..

[77] Chen H.H., Neutel J., Smith D., Heublein D., Burnett J. (2016). A first-in-human trial of a novel designer natriuretic peptide ZD100 in human hypertension. J. Am. Soc. Hypertension.

